# Complete Genome Sequence and Analysis of a ST573 Multidrug-Resistant Methicillin-Resistant *Staphylococcus aureus* SauR3 Clinical Isolate from Terengganu, Malaysia

**DOI:** 10.3390/pathogens12030502

**Published:** 2023-03-22

**Authors:** Esra’a I. Al-Trad, Ainal Mardziah Che Hamzah, Suat Moi Puah, Kek Heng Chua, Muhamad Zarul Hanifah, Qasim Ayub, Prasit Palittapongarnpim, Stephen M. Kwong, Ching Hoong Chew, Chew Chieng Yeo

**Affiliations:** 1 Centre for Research in Infectious Diseases and Biotechnology (CeRIDB), Faculty of Medicine, Universiti Sultan Zainal Abidin, Kuala Terengganu 20400, Malaysia; 2 Faculty of Health Sciences, Universiti Sultan Zainal Abidin, Kuala Nerus 21300, Malaysia; 3 Department of Biomedical Science, Faculty of Medicine, Universiti Malaya, Kuala Lumpur 50603, Malaysia; 4 Monash University Malaysia Genomics Facility, School of Science, Monash University, Bandar Sunway 47500, Malaysia; 5 Pornchai Matangkasombut Center for Microbial Genomics (CENMIG), Mahidol University, Bangkok 10400, Thailand; 6 Infectious Diseases & Microbiology, School of Medicine, Western Sydney University, Campbelltown 2560, Australia

**Keywords:** *Staphylococcus aureus* ST573, hybrid assembly, resistance genes, SCC*mec* element, plasmids, genomic islands, prophages

## Abstract

Methicillin-resistant *Staphylococcus aureus* (MRSA) is a World Health Organization-listed priority pathogen. Scarce genomic data are available for MRSA isolates from Malaysia. Here, we present the complete genome sequence of a multidrug-resistant MRSA strain SauR3, isolated from the blood of a 6-year-old patient hospitalized in Terengganu, Malaysia, in 2016. *S. aureus* SauR3 was resistant to five antimicrobial classes comprising nine antibiotics. The genome was sequenced on the Illumina and Oxford Nanopore platforms and hybrid assembly was performed to obtain its complete genome sequence. The SauR3 genome consists of a circular chromosome of 2,800,017 bp and three plasmids designated pSauR3-1 (42,928 bp), pSauR3-2 (3011 bp), and pSauR3-3 (2473 bp). SauR3 belongs to sequence type 573 (ST573), a rarely reported sequence type of the staphylococcal clonal complex 1 (CC1) lineage, and harbors a variant of the staphylococcal cassette chromosome *mec* (SCC*mec*) type V (5C2&5) element which also contains the *aac(6′)-aph(2″)* aminoglycoside-resistance genes. pSauR3-1 harbors several antibiotic resistance genes in a 14,095 bp genomic island (GI), previously reported in the chromosome of other staphylococci. pSauR3-2 is cryptic, whereas pSauR3-3 encodes the *ermC* gene that mediates inducible resistance to macrolide-lincosamide-streptogramin B (iMLS_B_). The SauR3 genome can potentially be used as a reference genome for other ST573 isolates.

## 1. Introduction

*Staphylococcus aureus* has been identified as an important pathogen in Malaysia, causing infections that range from mild cases, such as boils, to potentially lethal infections such as bacteremia [1,2]. The pathogenesis of *S. aureus* has been linked with several virulence factors such as enterotoxins, hemolysins, protein A, and toxic shock syndrome toxin [3]. Moreover, the World Health Organization (WHO) has listed *S. aureus* as one of the rapidly evolving bacteria that are able to develop resistance to a wide spectrum of antimicrobial classes such as β-lactams, aminoglycosides and macrolide-lincosamide-streptogramin B (MLS_B_) [4]. The prominent resistant strain of *S. aureus* is methicillin-resistant *S. aureus* (MRSA) which exhibits resistance to most β-lactam antibiotics and is often multidrug-resistant (MDR), being resistant to three or more classes of antimicrobials [5].

Resistance to methicillin is due to a target modification mechanism wherein the *mecA* gene produces modified penicillin-binding protein 2a (PBP2a), causing low affinity for β-lactam antibiotics. The *mecA* gene is carried by staphylococcal cassette chromosome *mec* (SCC*mec*), a mobile genetic element [6]. To date, various types of SCC*mec* elements have been described in staphylococci, with the most recent being SCC*mec* type XV [7,8]. Besides SCC*mec*, the MRSA genome contained various other mobile genetic elements such as bacteriophages, pathogenicity islands and plasmids. These mobile elements may contain various genes that are important for MRSA adaptability, pathogenicity and survival under certain harsh conditions, such as exposure to antimicrobials [9]. MRSA is prevalent in most Asian hospitals with country-to-country variability [10]. MRSA prevalence is estimated to range from 31.4% in China [11] to 14.9% in Malaysia, with most of these Malaysian MRSA isolates obtained from blood (24.6%) [12].

In the last decade, sequencing of the whole genomes of pathogens has led to in-depth understanding of the architecture and evolution of bacterial genomes [13]. Sequencing data also provide valuable information about clinical isolates including molecular epidemiological markers such as multilocus sequence types (MLST), clonal complex (CC) lineages, and carriage of antimicrobial resistance and virulence genes, which could be useful in clinical decision making and infection control. So far, there have been limited comprehensive studies that have been carried out in Malaysia concerning the genome’s architecture and comparative analysis of the clinical MRSA population. Recent reviews on the characteristics of MRSA isolates from Malaysian hospitals indicated the high incidence of the ST239-SCC*mec* III clone (also known as the Hungarian clone), but this appeared to have been replaced in recent years by the ST22-SCC*mec* IV clone (EMRSA-15) [14,15,16], a situation similar to that previously reported in Singapore [17]. In this paper, we present the complete genome sequence of a multidrug-resistant clinical MRSA isolate designated SauR3, which is of ST573, a rarely reported sequence type for which there has yet to be a complete genome sequence in the public databases. To our knowledge, there has only been a single report of an ST573 isolate from Malaysia, which was isolated from a nasal swab in 2008 [18]. This study presents the complete genome sequence of *S. aureus* SauR3, which was obtained from hybrid assembly of Illumina HiSeq and Oxford Nanopore Technologies (ONT) MinION reads. This enabled us to obtain an in-depth understanding of its genomic architecture, its resistance profile and virulence strategies in comparison with other MRSA isolates in the databases.

## 2. Results and Discussion

### 2.1. Background of the S. aureus SauR3 Isolate

*S. aureus* SauR3 was isolated from the blood of a six-year-old female patient who was admitted to the emergency unit of Hospital Sultanah Nur Zahirah (HSNZ) in Kuala Terengganu, Malaysia, in January 2016. The patient had a pre-existing history of Down’s syndrome with congenital hypothyroidism and a global developmental delay, and presented at the hospital with facial and preseptal cellulitis. The blood sample of the patient was obtained for culture, and she was hospitalized for six days, during which time she was treated with intravenous amoxicillin–clavulanic acid and chloramphenicol eyedrops along with ointment applied to both eyes. Following resolution of the symptoms of infection, the patient was discharged.

In the research laboratory, SauR3 was previously validated as MRSA by positive PCR reaction for the *nuc* and *mecA* genes. In an antibiotic susceptibility test targeting 26 antibiotics from 18 antimicrobial classes [5], SauR3 displayed resistance towards five antimicrobial classes that encompassed nine antibiotics, including β-lactams (penicillin, oxacillin, cefoxitin, cefoperazone), fluoroquinolones (ciproflaxin, moxifloxacin), macrolides (erythromycin), lincosamides (clindamycin), and aminoglycosides (gentamicin). SauR3 was thus classified as MDR according to the criteria recommended by the joint commission of the U.S. Center for Disease Control and Prevention (CDC) and the European Centre for Disease Prevention and Control (ECDC), i.e., it is resistant towards three or more classes of antimicrobials [19]. SauR3 also exhibited the inducible MLS_B_ (or known as inducible clindamycin resistance) phenotype using the D-test, meaning that the isolate could be induced to clindamycin resistance leading to treatment failure if the infected patient was administered clindamycin [20].

### 2.2. Genome Features and Molecular Typing of SauR3

Short-read sequencing using the Illumina HiSeq-PE150 platform generated 12,856,640 raw reads with a total of 1.9 Gbp raw data (approximately 300 × coverage), whereas long-read sequencing on the Oxford Nanopore MinION Mk1B platform generated 35,751 reads for a total of around 279 Mbp (97.1 × coverage). Hybrid assembly of the Illumina short paired-end reads and the Nanopore long reads resulted in the complete genome of SauR3 strain that consisted of a single circular 2,800,107 bp chromosome with a GC content of 32.8%, and which is similar to those of other sequenced *S. aureus* strains [21,22]. Three circular plasmids of 42,928 bp, 3011 bp and 2473 bp were also identified in the assembled genome, and these were designated pSauR3-1, pSauR3-2 and pSauR3-3, respectively. A total of 2595 coding regions, 60 tRNA genes, and 19 rRNA loci were identified in the SauR3 genome.

Analysis of the SauR3 sequence data revealed that SauR3 belonged to *spa* type t458, as had been previously determined by PCR [23], and sequence type 573 (ST573), a rare clone of clonal complex 1 (CC1) previously reported in Australia [24] and Taiwan [25]. To our knowledge, there has only been one report of a MRSA ST573 isolate from Malaysia, which was from a nasal swab of a patient in the orthopedic ward of the University of Malaya Medical Centre (UMMC) in 2008 [18]. UMMC is located in Kuala Lumpur, the capital city of Malaysia, which is approximately 445 km away from HSNZ in Kuala Terengganu. SauR3 carried a variant of the SCC*mec* type V (5C2&5), which will be presented in detail in Section 2.6.

### 2.3. Prediction of Antimicrobial Resistance Genes from the SauR3 Genome

A total of 15 antimicrobial resistance genes were detected in the assembled SauR3 genome using CARD and ResFinder (Table 1). Most of the resistance genes detected corresponded with the resistance phenotype of SauR3 except for fosfomycin, whereby SauR3 was susceptible despite the carriage of the *fosB* gene, which encodes a metallothioltransferase enzyme that inactivates fosfomycin. However, the *fosB* gene in SauR3 was 99% identical in sequence to the reference *fosB* gene in the CARD database (accession no. WP_000920237), and sequence alignment showed an asparagine to aspartate substitution at amino acid residue 123 of the FosB protein. Whether this mutation is involved in SauR3 showing fosfomycin susceptibility despite the presence of the *fosB* gene will require further investigation.

Three genes which encode efflux pumps of the major facilitator superfamily (MFS), namely, *norA*, *norC* and *sdrM* which have been implicated in resistance to fluoroquinolones, were detected in the SauR3 chromosome. Previous studies had also reported the roles of these efflux pumps in mediating resistance toward antiseptics and disinfectants [26,27]. Other resistance determinants that were found on the SauR3 chromosome were the *aac(6′)-aph(2″)* gene encoding the aminoglycoside acetyltransferase (which confers gentamicin resistance) that was located on the SCC*mec* element, and the *lmrS* gene that encodes an MFS transporter which may have a role in reducing the susceptibility of SauR3 towards macrolides and aminoglycosides [28,29], besides chloramphenicol and erythromycin [30].

**Table 1:** Antimicrobial resistance genes identified in the *S. aureus* SauR3 genome and its detected resistance phenotype.

Antimicrobial Class	Resistance Phenotype	Detected Resistance Phenotype *	Resistance Genotype	Nucleotide Coordinates ^‡^	Location of the Resistance Gene	Mechanism of Resistance
β-lactams	Penicillin	R	*blaZ* family	24,826–23,981	pSauR3-1	Antibiotic inactivation enzyme
	Cefoxitin, oxacillin, cefoperazone	R	*mecA*	1,987,326–1,985,317	Chromosomal/SCC*mec*	Antibiotic target alteration
Fluoroquinolones	Ciprofloxacin, moxifloxacin	R	*norA*, *norC*, *sdrM*	1,284,437–1,283,271 1,927,502–1,926,114 2,609,957–2,611,300	Chromosomal	MFS efflux pump
Macrolides	Erythromycin	R (iMLS_B_)	*ermC*	1407–2141	pSauR3-3	Antibiotic target alteration
			*lmrS*	2,612,195–2,613,637	Chromosomal	Efflux pump
			*msrA*	33,114–31,648	pSauR3-1	Antibiotic target protection
			*mphC*	31,549–30,650	pSauR3-1	Antibiotic inactivation enzyme
Lincosamide	Clindamycin	R (iMLS_B_)	*ermC*	1407–2141	pSauR3-3	Antibiotic target alteration
Aminoglycosides	Gentamicin	R	*aph(3′)-IIIa, aadE*	35,670–36,464	pSauR3-1	Antibiotic inactivation enzyme
			*aac(6′)Ie-aph(2″)Ia*	1,961,711–1,963,150	Chromosomal/SCC*mec*	Antibiotic inactivation enzyme
Fosfomycin	Fosfomycin	S	*fosB*	2,465,206–2,465,625	Chromosomal	Antibiotic inactivation enzyme
Atypical aminoglycoside	Streptothricin	ND	*SAT-4*	35,035–35,577	pSauR3-1	Antibiotic inactivation enzyme

* Resistance phenotype as detected by disk diffusion; R, resistant; S, susceptible; ND, not determined; iMLSB, inducible macrolide-lincosamide-streptogramin B (inducible clindamycin resistance) phenotype determined by D-test; MFS, major facilitator superfamily; ^‡^ Nucleotide coordinates are as in GenBank accession no. CP098727 for the SauR3 chromosome, CP098728 for pSauR3-1, and CP098730 for pSauR3-3.Plasmids harbored by SauR3 also contributed to its resistome. The *SAT-4* gene which was found on the 42.9 kb pSauR3-1, encodes streptothricin acetyltransferase and mediates streptothricin resistance. However, phenotypic susceptibility of SauR3 against streptothricin (member of nucleoside class) was not tested, as this compound was not in the ECDC-CDC recommended list of antimicrobials to be tested for staphylococci [19]. pSauR3-1 also encodes the aminoglycoside resistance genes, *aph(3′)-IIIa* (aminoglycoside *O*-phosphotransferase) and *aadE* (aminoglycoside nucleotidyltransferase), which likely mediates gentamicin resistance in SauR3, and the *msrA* and *mphC* genes that confer macrolide and streptogramin B resistance [31]. The smallest plasmid in SauR3, the 2473 bp pSauR3-3, contains the *ermC* gene that encodes 23S ribosomal RNA methyltransferase and mediates resistance to macrolides and lincosamides [32].

### 2.4. Prediction of Virulence Genes in the SauR3 Genome

A total of 83 virulence determinants categorized into five virulence classes (Supplementary Table S1) were predicted in the SauR3 chromosome and distributed as follows: 15 genes that mediate adherence, eight genes that encode various enzymes which promote colonization, 18 genes that play various roles in immune evasion (15 of which are essential for capsule production), 10 genes that encode type VII secretion system proteins and finally, 32 genes that encode various toxins. Of these 32 toxin-coding genes, 14 encode enterotoxins such as enterotoxin-like P, which have been identified as a major risk factor for *S. aureus* bacteremia [33,34]. In addition, two leukocidin toxins were identified in SauR3, designated LukG and LukH, which target and lyse macrophages, monocytes, neutrophils, and dendritic cells, and thus further contribute to its pathogenesis. An additional virulence gene, *ednA*, was predicted on pSauR3-1, which encodes epidermal cell differentiation inhibitor A that facilitates the hematogenous spread of *S. aureus* to deeper tissues [35]. Thus, besides being an MDR strain, SauR3 also harbors numerous virulence genes, a finding that is similar to many other MRSA strains that have been reported to carry various virulence determinants that are crucial for the success of this bacterium in the human host [36,37,38].

### 2.5. Phylogenetic Analysis of SauR3

**Figure 1:**
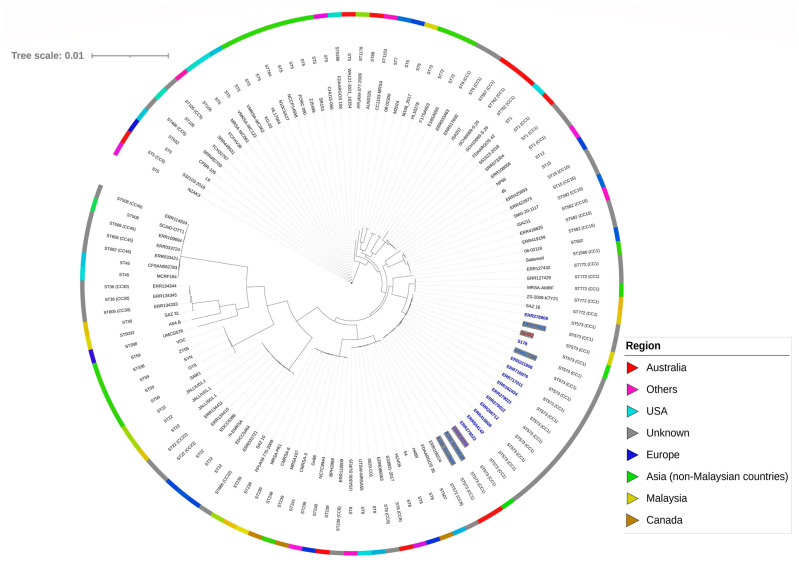
Core genome maximum-likelihood phylogenetic tree of MRSA SauR3 in comparison with other *S. aureus* isolates. The MRSA SauR3 core genome comprises 1635 core genes from a total of 8215 genes. ST indicates sequence type. CC indicates clonal complex. The ST573 strains are shown in dark blue fonts with SauR3 in red, while the ST573 MRSA strains are highlighted in gray. The list of genomes that were used to construct the phylogenetic tree is in Supplementary Table S2.

**Figure 2:**
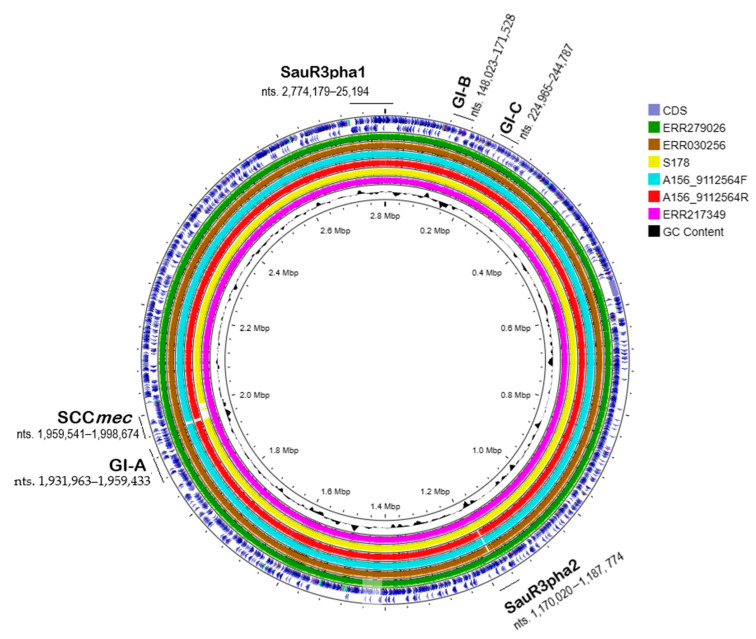
Comparison of the *S. aureus* SauR3 genome sequence using CGView with genomes of closely related ST573 MRSA/MSSA strains, i.e., MRSA ERR279026 (PubMLST id: 15635), MRSA ERR030256 (PubMLST id: 7808), MRSA strain A156_9112564F (PubMLST id: 36174), MRSA strain A156_9112564R (PubMLST id: 36183), MRSA ERR217349 strain (PubMLST id:10358), and MSSA S178 (PubMLST id: 43067) strains. The SauR3 genome was used as the main reference genome for the CGView BLASTN comparison. From outside to the center, rings 1 and 2 show coding sequences for the SauR3 forward and reverse strands; ring 3 shows the ERR279026 genome (in green), ring 4 shows ERR030256 (in brown), ring 5 shows A156_9112564F (in sky blue), ring 6 shows A156_9112564R (in red), ring 7 shows S178 (in yellow), ring 8 shows ERR217349 (in purple) and finally, the innermost ring 9 shows a plot of the GC content. The locations of the genomic islands, prophages, and the SCC*mec* element identified in the SauR3 genome are labelled, along with their nucleotide coordinates.

Phylogenetic analysis of the core genome of SauR3 with a sample of various *S. aureus* genomes showed that SauR3 clustered together with isolates that were typed as ST573 and were deposited in PubMLST (Figure 1; Supplementary Table S2). These included both MRSA and MSSA isolates. SauR3 was most closely related to *S. aureus* ERR279026, which is also an MRSA and ST573 isolate but with an unknown isolate origin in which the genome was submitted in the PubMLST database (PubMLST id: 15635). The average nucleotide identity (ANI) between the SauR3 and ERR279026 genomes was 99.94%. ST573 belongs to the CC1 lineage and interestingly was found to cluster next to ST772 clones, which were also CC1. ST573 is a rarely reported clone, whereas ST772, which was first isolated in India and is prevalent in Asia, has now been reported worldwide [39,40]. The ST573 isolates were all closely related, regardless of whether they are categorized as MRSA or MSSA, with ANI values of not less than 99.72% among them. Furthermore, the MRSA isolates of ST573 all harbored similar SCC*mec* type V elements (see Section 2.6 for details), strongly inferring that the MRSA ST573 lineage likely came about through acquisition of the SCC*mec* type V element in a MSSA ST573 clone. Likewise, the European multidrug-resistant CC1-MRSA-IV clone (of ST1 and *spa* type t127) had emerged from a methicillin-susceptible lineage [41], and the Chinese ST22-MRSA-V (of *spa* type t309) clone was derived from a ST22 MSSA clone [42]. Comparison of some of these ST573 genomes using CGView showed their high degree of similarities (Figure 2), and it is clear that the main differences of the MSSA isolate, *S. aureus* S178 (PubMLST id: 43067) from the other ST573 MRSA isolates lies in the absence of the SCC*mec* type V element. Similarities were also observed in the carriage of genomic islands and prophages in these ST573 isolates (Figure 2), further details of which are presented in Section 2.7 and Section 2.8.

### 2.6. The SCCmec Element in SauR3

**Figure 3:**
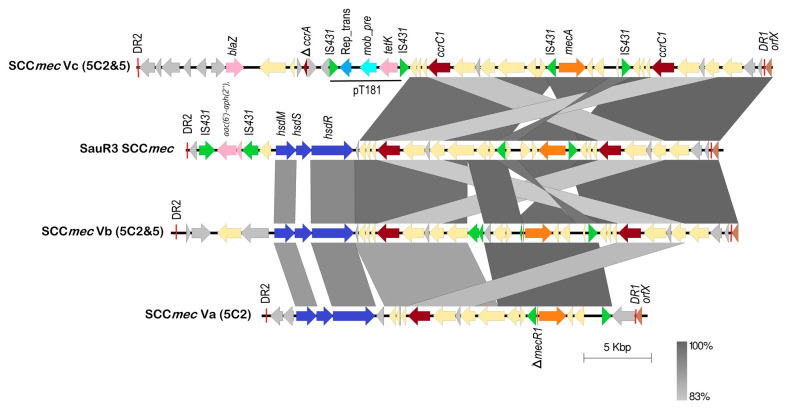
Comparison of the genetic organization of the SCC*mec* element of SauR3 with SCC*mec* type Va of *S. aureus* JCSC3624 (WIS) (accession no. AB121219; 28,612 bp), SCC*mec* type Vb of *S. aureus* TSGH17 (accession no. AB512767; 42,083 bp) and SCC*mec* type Vc of *S. aureus* JCSC6944 (accession no. AB505629; 47,212 bp). The *mecA* methicillin resistance gene and its associated *mecR* regulator (in truncated form and labeled as Δ*mecR1*) are depicted as orange-colored arrows; the site-specific recombinase genes (*ccrC1 and* truncated *ccrA*, labeled as Δ*ccrA*) are shown as dark red arrows; the *orfX* gene is colored brown; IS*431*-encoded transposases are colored green; type I restriction modification genes (*hsdR*, *hsdS* and *hsdM*) are depicted as dark blue arrows. DR1 and DR2 indicate direct repeats that flank SCC*mec* elements; other antimicrobial resistance genes are shown as pink arrows and include *aac(6′)-aph(2″),* aminoglycoside resistance genes; *tetK*, a tetracycline resistance gene; and *blaZ*, a gene encoding β-lactamase. The Rep_trans domain replicase and the plasmid mobilization gene *mob_pre* of plasmid pT181 found in SCC*mec* Vc (5C2&5) are indicated in turquoise and light blue arrows, respectively. Hypothetical protein-coding sequences are depicted as grey arrows, while other genes that encode proteins with known functional homologs are shown as light yellow arrows.

The *mecA* gene which conferred methicillin/oxacillin resistance was found in a 39,133 bp SCC*mec* type V (5C2&5) variant in SauR3 (spanning nts. 1,959,541–1,998,674). This size is in accordance with that of other SCC*mec* type V elements previously described in other *S. aureus* isolates, which range in length from 24 to 39 kb [43]. The SCC*mec* element in SauR3 showed similarities with several SCC*mec* type V subtypes (Figure 3), namely SCC*mec* type Va of *S. aureus* strain JCSC3624 (WIS) (accession no. AB121219; 28,612 bp), SCC*mec* type Vb of *S. aureus* strain TSGH17 (AB512767; 42,083 bp) and SCC*mec* type Vc of *S. aureus* strain JCSC6944 (AB505629; 47,212 bp), all of which were listed in the International Working Group on the Staphylococcal Cassette Chromosome Elements (IWG-SCC) website (https://www.sccmec.org/index.php/en/sccmmcc-list-smn-en/current-sccmmcc-types accessed on 1 December 2022) [7].

The *orfX* gene (also called *rlmH*) and the SCC*mec* insertion site, which is positioned at the 3′ end of this gene, of the SauR3-encoded SCC*mec* were 100% identical to those observed in SCC*mec* type Vb (5C2&5) and SCC*mec* type Vc (5C2&5). This gene is conserved among all staphylococci but its function has not yet been fully determined, and the SCC*mec* elements are integrated into the chromosome at a specific site located at the end of this gene [44].

The *mecA* gene, which confers resistance to methicillin and other β-lactams, is located downstream of *orfX* and is flanked by IS*431*, divergently orientated with respect to each other. Interestingly, the orientation of the *mecA* gene in the SauR3 SCC*mec* element was inverted when compared to the three SCC*mec* type V subtypes (Figure 3). Such an inverted arrangement of the *mecA* gene has been previously observed in the SCC*mec* type V of *S. aureus* WIS strain [43] and SCC*mec* type X of *S. aureus* JCSC6945 strain [43,45]. The *mecA*-associated regulatory gene, *mecR1*, was present in truncated form in SCC*mec* type Va (5C2) and SCC*mec* type Vb (5C2&5), but was not observed in the SauR3-encoded SCC*mec* and SCC*mec* type Vc (5C2&5). However, BLASTN analysis of the region indicated the presence of the truncated *mecR1* gene in both the SauR3 SCC*mec* and type Vc (5C2&5), inferring that the absence of the gene was overlooked during gene prediction and annotation, as had been previously described [46].

The SauR3 SCC*mec* element carried two *ccrC1* genes (i.e., allele 2 and allele 8) that were identical to those found in subtypes Vb and Vc, whereas subtype Va only harbored one *ccrC1* gene (allele 2). The two *ccrC1* genes shared 83% sequence identity with each other, and both encode site-specific recombinases which play an essential role in the excision and integration of the SCC*mec* element [47]. The *ccrC1* (allele 8) is located in the region between the *orfX* and *mecA* genes, while *ccrC1* (allele 2) is found downstream of the *mecA* gene.

The SauR3 SCC*mec* element also encodes for a full set of *hsdR*, *hsdS* and *hsdM* type I restriction–modification system, which was also found in subtypes Va and Vb, but not in subtype Vc (Figure 2). The *hsdR* and *hsdM* genes of the SauR3 SCC*mec* element were identical in sequence to the corresponding genes in SCC*mec* types Va and Vb, whereas *hsdS* found in the same two SCC*mec* subtypes shared 35% sequence identity. Previous studies have described major sequence variation in *hsdS* genes between different *S. aureus* lineages [48,49,50]. HsdS is the specificity subunit of the three-component Hsd system, and it requires interaction with the HsdM modification subunit. Once bound, the HsdS-HsdM complex can interact with the HsdR restriction subunit, which then exerts exonuclease activity at specific recognition sites, depending on the methylation state of the target sequence [51]. It is thus interesting to note the relative sequence dissimilarities of the *hsdS* component of the *hsdMSR* cluster in the SauR3-encoded SCC*mec* type V, and in subtypes Va and Vb. Carriage of type I restriction-modification system proteins may protect the host from invading genomes (such as bacteriophages), as these protein complexes can destroy foreign DNA introduced by an infectious agent [52]. The significance of the *hsdS* sequence divergence is unknown at this juncture.

It is worthy to note the SauR3-encoded SCC*mec* element contained the aminoglycoside resistance genes, *aac(6′)-aph(2″),* that were flanked by divergently oriented IS*431* elements. This was also found in the SCC*mec* of *S. aureus* ERR279026 and ERR217349, but was not observed in other SCC*mec* type V elements, particularly from other ST573 isolates. Nevertheless, SCC*mec* elements are well known to contain other additional resistance determinants besides *mecA* [53,54,55]. For example, an integration of the pT181 tetracycline resistance plasmid was observed in the SCC*mec* Vc subtype, which also contained the *blaZ* β-lactamase gene (Figure 3).

### 2.7. Genomic Islands in SauR3

**Figure 4:**
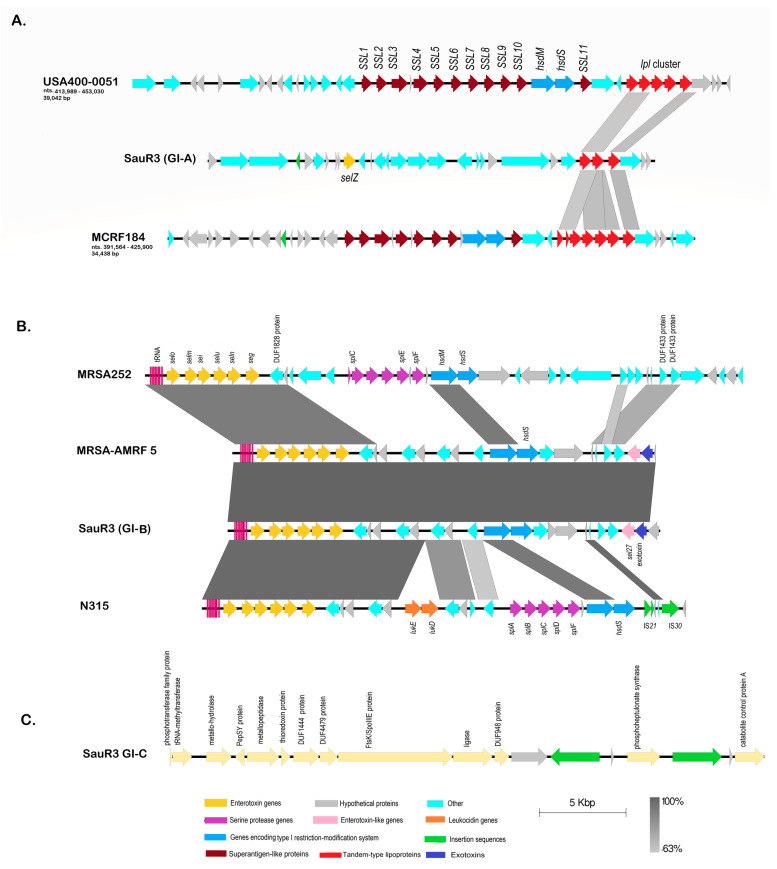
Genetic organization of genomic islands (GIs) predicted in the *S. aureus* SauR3 genome using IslandViewer 4. (**A**) Linear map and comparison of genetic structures of the SauR3 GI-A, with the *S. aureus* vSaα pathogenicity island found in *S. aureus* MCRF strain (accession no. NZ_CP014791) and MRSA strain USA400-0051 (accession no. NZ_CP019574.1). (**B**) Linear map and comparison of genetic structures of the SauR3 GI-B with the *S. aureus* vSaβ pathogenicity island found in *S. aureus* N315 (accession no. NC_002745.2), MRSA-AMRF 5 strain (NZ_CP062467), and *S. aureus* MRSA252 (accession no. NC_002952.2). (**C**) Linear map of the predicted SauR3 GI-C island.

IslandViewer 4 predicted three genomic islands from the SauR3 genome, designated GI-A, GI-B and GI-C (Figure 4). GI-A is 27,470 bp in length and is located at nts. 1,931,963–1,959,433 of the SauR3 genome, adjacent to the SCC*mec* element. GI-A contains a tandem of lipoprotein-encoding genes (LC189_09420, LC189_09425, and LC189_09430) as well as the *selZ* gene encoding enterotoxin type Z (Figure 4A). The existence of lipoprotein gene cluster (*lpl*) on a genomic island in MRSA has been described [56,57], but their contribution to virulence is not clear. One study reported the ability of lipoproteins to stimulate and enhance host cell invasion, which may facilitate its spread in the host and increase pathogenicity [58]. The *lpl* gene cluster of GI-A showed around 60% nucleotide sequence identity to the *lpl* cluster found on the *v*Saα pathogenicity island that was previously reported in *S. aureus* strain MCRF (accession no. NZ_CP01479) [59] and MRSA strain USA400-0051 (accession no. NZ_CP019574.1) [60]. However, no other sequence similarities with other regions of the *v*Saα pathogenicity island, including genes encoding the superantigen-like proteins, were observed (Figure 4A). As was shown in the CGView comparison (Figure 2), intact GI-A was found in the genomes of all ST573 isolates that were analyzed.

GI-B from SauR3 is 23,505 bp in length, spanning nts. 148,023–171,528, and is located downstream of a cluster of eight tRNA genes (from tRNA-Met at nts. 147,067–147,710 to tRNA-Ser at nts. 147,721–147,809). GI-B contains a cluster of six enterotoxin genes, i.e., *seo*, *sem*, *sei*, *seu*, *sen*, and *seg*, along with an enterotoxin-like gene, *sel27*, and an exotoxin-encoding gene (locus tag LC189_01045). The latter two genes are adjacent to each other and located at the opposite end of the GI-B island from the enterotoxin gene cluster (Figure 4B). GI-B also contains the *hsdMS* type I restriction-modification genes. The cluster of tRNA genes and the six enterotoxin genes along with the *hsdM* gene in GI-B are highly identical in sequence and organization to the *v*Saβ pathogenicity island previously observed in *S. aureus* N315 (NC_002745.2), MRSA-AMRF 5 (NZ_CP062467) and *S. aureus* MRSA252 (NC_002952.2) isolates [49] (Figure 4B), and more recently in MRSA ST9 isolates from China and Germany [61]. However, the *hsdS* gene in GI-B was unrelated to the *hsdS* in the *v*Saβ island of MRSA252 and N315, but identical to the *hsdS* in the GI of MRSA-AMRF 5. Interestingly, the serine protease or *spl* gene cluster found in *v*Saβ of MRSA252 and N315 was absent in both GI-B and the GI of MRSA-AMRF 5. The role of the Spl serine proteases in virulence is not fully determined, but these enzymes are immunogenic and can induce allergic reactions in individuals infected with *S. aureus,* and they have also been shown to be involved in colonization [62,63]. GI-B was found in other related ST573 MRSA/MSSA related strains (Figure 2).

The third putative genomic island predicted in SauR3, GI-C, was 19,823 bp in length and spanned nts. 224,965–244,787 (Figure 4C). No apparent virulence or resistance genes were found in GI-C, which is bounded by a *ccpA* gene encoding catabolite control protein A and a *tmbB* gene encoding tRNA (guanosine-N7)-methyltransferase with two IS*1182* transposases in the predicted island. As was seen for GI-B, GI-C was also identified in the other ST573 MRSA/MSSA strains (Figure 2). However, no similarities with known genomic/pathogenicity islands were detected for GI-C.

### 2.8. Prophages in SauR3

**Figure 5:**
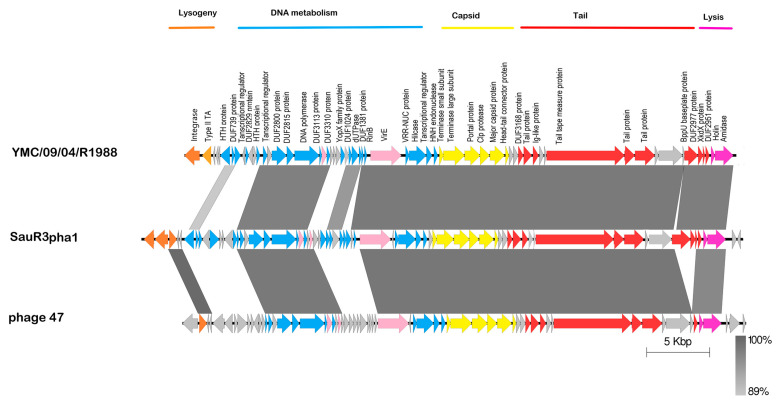
Comparison of the genetic organization of the predicted SauR3-pha1 phage with the MRSA phage YMC/09/04/R1988 (accession no. NC_022758.1) and *Staphylococcus* phage 47 (NC_007054). The genes were color-coded into different regions based on their putative functionalities, i.e., lysogeny (in orange), DNA metabolism (blue), capsid (yellow), phage tail proteins (red), and lysis (pink). Putative virulence-associated genes are indicated in light pink. Hypothetical proteins are presented in grey. Abbreviations: VirE, virulence protein E; type II TA, type II toxin-antitoxin system; *sph*, sphingomyelin phosphodiesterase.

PHASTER identified two potential prophages in the SauR3 genome. The first was an intact phage designated SauR3-pha1 (51,124 bp in length; located at nts. 2,774,179–2,800,107 and continued at nts. 1–25,194) and the other was tagged by PHASTER as an incomplete or partial phage designated SauR3-pha2 (17,754 bp in length, located at nts. 1,170,020–1,187,774), as indicated in Figure 2. The SauR3-pha1 phage showed similarities to the MRSA phage YMC/09/04/R1988 (44,459 bp; accession no. NC_022758.1) and a staphylococcal phage 47 (44,777 bp; accession no. NC_007054), both of which belonged to the family Siphoviridae and showed a broad host range [64]. Comparison of these phage genomes showed high sequence similarities in the capsid and tail formation as well as the lysis regions of the phages (Figure 5). However, the lysogeny region of the SauR3-pha1 appeared to be less related to both YMC/09/04/R1988 and 47 phages, except for an ATPase and two hypothetical proteins which were 100% identical to those in phage 47 (Figure 5). Some differences were also observed in the DNA processing/metabolism region of the phages (Figure 5). SauR3-pha1 was observed in all the ST573 genomes.

The incomplete SauR3-pha2 phage had a GC content of 29.8% and comprised 21 protein coding sequences. SauR3-pha2 showed sequence identities with only short stretches (the longest being 924 bp) of *Staphylococcus* phage StauST398-5 (43,301 bp; accession no. NC_023500.1). SauR3-pha2 is likely a partial phage, as it lacks lysis and terminase genes and could possibly be a remnant of a past incomplete excision event. Temperate bacteriophages are one of the drivers of the evolution of bacterial genomes, and prophages have been shown to play a key role in the adaptation of the initially pig-borne ST398 clone of *S. aureus* to humans [65].

### 2.9. Plasmids in SauR3

Three distinct plasmids could be identified from the assembled SauR3 genome, and these are designated (based on size from larger to smaller) pSauR3-1 (42,928 bp), pSauR3-2 (3011 bp) and pSauR3-3 (2473 bp). The largest plasmid, pSauR3-1, carries various antimicrobial resistance genes including the *bla* operon for penicillin resistance, the *mphC* and *msrA* genes for macrolide and streptogramin resistance, the *SAT-4* gene for streptothricin resistance, and the *aph(3″)-IIIa* and *aadE* genes for aminoglycoside resistance. Genes that mediate resistance to cadmium ions, i.e., *cadDX*, were also identified in pSauR3-1. Apart from resistance genes, pSauR3-1 also harbored the *ednA* and *se1* virulence genes that encode epidermal cell differentiation inhibitor A and an enterotoxin, respectively. Intriguingly, pSauR3-1 contained two full-length replication initiation genes, *rep19* and *rep20*, both of which belonged to the RepA_N family of replicases. In addition, a partial *rep21* replicase belonging to the Rep_1 family was also identified. Multiple-replicon plasmids were previously documented [66,67,68] and are of special importance because their prevalence may suggest their tendency to circulate across various hosts, making them vectors for the transmission of undesired traits such as antibiotic resistance and virulence [67,69,70,71]. A type I toxin-antitoxin system (the Fst family toxin) was also observed; this has been previously reported in several staphylococcal plasmids [72] and could play a crucial role in the stabilization and persistence of the plasmid. Apparently, the Fst toxin observed in pSauR3-1 backbone was overlooked from the annotation of both pJSA01 and EDINA plasmids. Fst is a small protein (around 30 amino acids) and in general, small protein-coding genes are a challenge for genome annotation and could easily be overlooked [73,74].

Although no conjugative or mobilization-related genes were found in pSauR3-1, the plasmid carries *oriT* sequences that are similar to pWBG749-encoded *oriT*-OT49 and *oriT*-OTUNa, subtypes which are known pWBG749 *oriT*-mimics that enabled the plasmid to be mobilizable if it coexists with a pWBG749-type plasmid [75]. In other words, dissemination of this multidrug-resistant virulence plasmid among staphylococcal clinical isolates is possible. This finding, i.e., the existence of several *oriT*-mimics on a given plasmid, coincides with a previous report of three copies of pWBG749 *oriT*-mimics being detected on pWBG744 (27,268 bp) and pWBG762 (54,023 bp) plasmids of *S. aureus* [75], which may increase their transmissibility.

**Figure 6:**
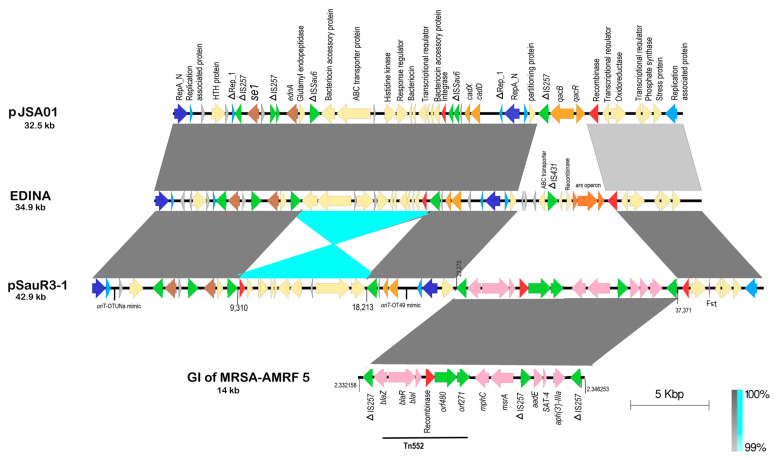
Sequence comparison between pSauR3-1 plasmid with the *S. aureus* E-1-encoded EDINA plasmid and *S. aureus* pJSA01 plasmid. The pSauR3-1 inverted region relative to plasmids EDINA and pJSA01 is highlighted in light blue colour (positioned at nts. 9310 to 18,213). A 14 kb fragment positioned in pSauR3-1plasmid at nts. 23,270 to 37,371 was found to be identical to an antimicrobial-resistant GI of MRSA-AMRF (positined at nts. 2,332,158 to 2,346,253 of the MRSA-AMRF genome). *qacA/qacR* encodes quaternary ammonium *compounds* resistance genes; p*271* encodes ATP-binding protein; p*480* encodes a transposase; *blaZ* encodes β-lactamase; *blaR* and *blaI* are β-lactamase regulatory genes; IS*257*/IS*431*/IS*Sau6* are insertion sequences; *cadDX*, cadmium resistance genes; *se1*, enterotoxin gene; RepA_N/Rep_1, replication conserved domains; *ednA*, epidermal cell differentiation inhibitor A gene; Fst, toxin protein; *SAT-4*, streptothricin resistance gene; *mphC*, macrolide resistance gene; *msrA*, macrolide and streptogramin B resistance genes; *aadE/aph(3′)-IIIa*, aminoglycoside resistance genes.

BLASTN analysis showed that pSauR3-1 is 99% identical to the *S. aureus* E-1-encoded plasmid EDINA (34,986 bp; accession number AP003089) and *S. aureus* plasmid pJSA01 (32,580 bp; accession number AP014922) (Figure 6). This similarity spans an approximately 23 kb region from the RepA_N replicase (LC189_013745) to the second RepA_N replicase (LC189_013880) and its corresponding partitioning protein (LC189_013885), and includes the *cadDX* cadmium resistance genes and the *ednA* and *se1* virulence genes (Figure 6). However, within this region, an inversion of approximately 9 kb was seen in pSauR3-1 (from nts. 9310 to 18,213) when compared to plasmids EDINA and pJAS01, and this region contained putative bacteriocin-like genes and an integrase/recombinase-encoded gene, and is flanked by partial IS*Sau6* elements. The presence of the integrase/recombinase gene and IS*Sau6* could account for the inversion, with IS*256* being previously reported to mediate an inversion of nearly 1 Mbp genomic region in the MRSA OC8 strain [76]. IS elements are known to facilitate the occurrence of reciprocal recombination events causing the region in between these elements to invert or flip [77]. Beside IS elements, integrase/recombinase can also facilitate DNA rearrangements such as inversion events [78].

Interestingly, an approximately 14 kb region that spans nts. 23,270–37,371 of pSauR3-1 showed no similarities with either the EDINA or pJAS01 plasmids, but rather was identical in sequence to a GI that was previously identified in *S. aureus* strain MRSA-AMRF5 from India (accession no. NZ_CP062467.1; GI positioned from nts. 2,332,158–2,346,252) (Figure 6). This GI consisted of a full-length Tn*552* which contained the *bla* operon encoding the *blaZ* β-lactamase and its associated regulatory genes, *blaR* and *blaI*. The island is flanked by partial IS*257* elements. Downstream of Tn*552*, several resistance genes (*mphC*, *msrA*, *SAT-4*, *aadE-aph(3″)-IIIa*) were found (Figure 6). This finding indicates that the antimicrobial resistance GI had likely inserted into an ancestral plasmid of pSauR3-1, and this insertion could possibly be mediated by IS*257*. Thus, insertion of this GI was a significant contributor to the emergence of a multidrug resistance plasmid in pSauR3-1, as both the EDINA and pJAS01 plasmid do not harbor any antimicrobial resistance genes (except the *qacBR* biocide resistance genes found only on pJAS01).

**Figure 7:**
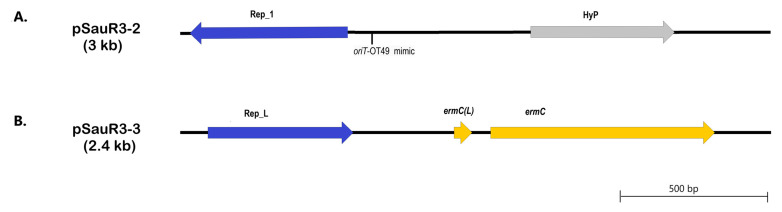
Linear genetic maps of the *S. aureus* SauR3-encoded pSauR3-2 plasmid (**A**) and pSauR3-3 plasmid (**B**). Rep_1 and RepL, replicase conserved domains; HyP, hypothetical protein; *ermC*, 23S rRNA methyltransferase gene that mediates MLSB resistance; *ermC(L)*, 23S rRNA methyltransferase *leader peptide*.

The other two plasmids in SauR3 are small plasmids, with pSauR3-2 being 3011 bp in size and pSauR3-3 being 2473 bp in size. The pSauR3-2 plasmid is cryptic, only encoding two genes: one for the *rep21* replicase of the Rep_1 family, and the other being a hypothetical protein (Figure 7A). The *rep21* replicase of pSauR3-2 shared 56% amino acid sequence identity with the Rep_1 replicase of *S. aureus* pUB110 plasmid which had been shown to have relaxase potential [79], thus suggesting that the pSauR3-2-encoded replicase could possibly function as a replicative relaxase, enabling the transfer of the plasmid as was previously described in several staphylococcal plasmids [80,81]. Further BLASTN searches revealed that pSauR3-2 also carried a sequence similar to that of pWBG749 *oriT*-OT49. Hence, the presence of two potential mobilization determinants in pSauR3-2 may facilitate its transfer if it happens to co-exist with compatible conjugative plasmids in the host.

The other small plasmid, pSauR3-3 also harbored two genes: the *rep10*-encoded replicase of the RepL domain and *ermC,* which encode a 23S rRNA methyltransferase gene (Figure 7B) that mediates resistance to MLS_B_ antibiotics. *S. aureus* SauR3 displayed an inducible MLS_B_ phenotype, and this is likely due to the presence of the *ermC* leader peptide coding sequence, designated *ermC(L)*, directly upstream of *ermC* [82] in pSauR3-3 (Figure 7B). pSauR3-3 is identical to the pUSA05-1-SUR11 plasmid (2473 bp; accession no. CP014405) and numerous other 2.4 kb staphylococcal plasmids. The Rep_L plasmids are known to be prevalent in staphylococci [83,84,85].

## 3. Concluding Remarks

Here, we report the complete genome sequence and analysis of a ST573 multidrug resistant, methicillin-resistant *S. aureus* strain SauR3, a hospital-acquired clinical isolate from Terengganu, Malaysia. ST573 strains, which are part of the Clonal Complex 1 (CC1) lineage, are rarely reported. Comparison of genomes of ST573 *S. aureus* isolates in the PubMLST database showed that they were both MRSA as well as MSSA, and were closely related, with ANI values of >99.7%. The genome of SauR3 harbored a variant of SCC*mec* type V (5C2&5) which also encodes the *aac(6′)-aph(2″)* aminoglycoside resistance genes. The other ST573 MRSA isolates analyzed also harbored similar variants of the SCC*mec* type V (5C2&5) element but without the aminoglycoside resistance genes. This SCC*mec* type V element was absent in the ST573 MSSA isolates, strongly implying that the MRSA isolates came about through the acquisition of the SCC*mec* element. Novel genomic islands with regions of similarities to known staphylococcal *v*Saα and *v*Saβ pathogenicity islands were identified in SauR3. SauR3 also harbored a 42.9 kb plasmid pSauR3-1, which was inserted with a multidrug resistance genomic island of about 14 kb that was previously discovered in the chromosome of an MRSA isolate from India. Two smaller plasmids were also identified in the SauR3 genome; pSauR3-2 was cryptic while pSauR3-3 harbored the *ermC* gene that mediated the iMLS_B_ phenotype. This work presented a detailed analysis of the complete genome sequence of a clinical ST573 *S. aureus*, which is one of the rarely reported sequence types of *S. aureus*, and could thus serve as a reference sequence for other ST573 strains.

## 4. Materials and Methods

### 4.1. Ethical Approval

The SauR3 clinical isolate and patient medical record were collected from Hospital Sultanah Nur Zahirah (HSNZ) with the approval from the Malaysian National Medical Research Registry and Medical Research and Ethics Committee (NMRR-MREC) of the Ministry of Health Malaysia, with approval number: NMRR-15-2369-28130 (IIR).

### 4.2. Growth of S. aureus SauR3

*S. aureus* SauR3 was originally isolated from the blood of a patient warded at HSNZ in January 2016. The isolate was stored as frozen glycerol archival stock at −80 °C in the university’s microbiology laboratory. For this study, a loopful of the frozen glycerol stock of *S. aureus* SauR3 was inoculated in mannitol salt agar, grown overnight at 37 °C and then subcultured on Miller’s Luria Bertani agar (Oxoid Ltd., Basingstoke, UK). The strain was validated as MRSA by PCR of *mecA* and *nuc* genes using previously described protocols [5].

### 4.3. Genomic DNA (gDNA) Extraction

SauR3 was one of the isolates that was sequenced in our MDR-MRSA genome sequencing project. The total genomic DNA (gDNA) was extracted using a phenol/chloroform extraction protocol with modification by using 25 mL of log phase bacterial culture [86]. The bacterial pellet was collected through centrifugation and resuspended in a buffer containing 0.15 M NaCl, 0.1 M EDTA and 2 mg/mL of lysostaphin (Sigma-Aldrich, Saint Louis, Missouri, USA), followed by incubation at 37 °C for 30 min. After that, lysis buffer (1% SDS, 0.1 M NaCl, 0.1 M EDTA) supplemented with 250 µg/mL proteinase K was added, and the mixture was incubated at 60 °C for 10 min. The digested lysate was purified with an equal amount of phenol/chloroform, and high-molecular-weight DNA was recovered from the resulting supernatant by isopropanol precipitation followed by triplicate washing steps with 70% ethanol. The quality of the extracted gDNA was evaluated using an Implen Nanophotometer^®^ spectrophotometer (Implen, Munich, Germany) and a Qubit 4 fluorometer (Thermo Fisher Scientific, Waltham, MA, USA), and visualized under ultraviolet transillumination following 1% (*w*/*v*) agarose gel electrophoresis.

### 4.4. Genome Sequencing, Assembly and Annotation

Whole genome sequencing was carried out using the Illumina platform with a paired-end sequencing strategy (HiSeq-PE150) by a commercial service provider (Novogene Co., Ltd., Singapore). Quality inspection was performed using FastQC v0.11.8 and MultiQC v1.7 [87]. Sequencing using the Oxford Nanopore *MinION* (Oxford Nanopore Technologies, Oxford, UK) platform was performed at the Monash University, Malaysia’s Genomics Facility. Briefly, high quality genomic DNA was subjected to Ligation Sequencing Kit SQK-LSK109 library preparation based on the manufacturer’s protocol. The DNA library was then sequenced using a MinION R10.3 flow cell on a MinION Mk1B sequencing device (Oxford Nanopore Technologies). The quality of the reads was determined using CheckM 1.2.0 (https://github.com/Ecogenomics/CheckM accessed on 1 September 2022). Base calling was conducted using Guppy v3.2.10 using a high accuracy configuration and configured to include trimming adapter sequences.

Hybrid assembly of the Illumina and Nanopore sequence data was performed using Unicycler v0.5.0 [88]. The assembled genome sequence was annotated using PROKKA [89] and the NCBI Prokaryotic Genome Annotation Pipeline (PGAP) [90] upon submission to GenBank.

### 4.5. Computational Genome Analysis

MLST was determined using PubMLST at https://pubmlst.org/ (accessed on 1 November 2022) [91]. SCC*mec* was identified using SCC*mec* Finder 1.2 at the Center for Genomic Epidemiology (https://cge.food.dtu.dk/services/SCCmecFinder/ accessed on 1 December 2022) [92,93] and the International Working Group on the Staphylococcal Cassette Chromosome elements (IWC-SCC) website at https://www.sccmec.org/index.php/en/ (accessed on 1 December 2022). The antimicrobial resistance genes were identified using the Comprehensive Antibiotic Resistance Database (CARD) [94] and ResFinder 4.1 (at https://cge.food.dtu.dk/services/ResFinder/ accessed on 1 November 2022). Virulence factors were identified using VFanalyzer, available in the Virulence Factors Database (VFDB) at http://www.mgc.ac.cn/VFs/main.htm (accessed on 1 November 2022) [95,96]. Phage Search Tool Enhanced release (PHASTER, at https://phaster.ca/ accessed on 1 December 2022) [97,98] was used to identify potential prophages in the genome sequence. IslandViewer 4 at https://www.pathogenomics.sfu.ca/islandviewer (accessed on 1 December 2022) was used to predict genomic islands (GIs) [99]. Comparative genome analysis was carried out using the NCBI BLAST+ tool kit available from (ftp://ftp.ncbi.nlm.nih.gov/blast/executables/blast+/LATEST/ accessed on 30 December 2022) then visualized by EasyFig 2.1 (http://mjsull.github.io/Easyfig/ accessed on 30 December 2022). Genome comparisons were also performed using CGView.js at Proksee (https://proksee.ca/ accessed on 30 December 2022). PlasmidFinder 2.1, available at the Center for Genomic Epidemiology (https://cge.food.dtu.dk/services/PlasmidFinder/ accessed on 15 November 2022), was used to identify and type plasmid-encoded replicases [92,93].

### 4.6. Phylogenetic Analysis

The *S. aureus* SauR3 phylogenetic tree was constructed by comparing with 118 assembled strains available either on GenBank (https://www.ncbi.nlm.nih.gov/data-hub/genome/?taxon=1280 accessed on 1 February 2023) or PubMLST (https://pubmlst.org accessed on 1 February 2023). This list includes all the ST573 genomes and related ST772 genomes available in PubMLST on 1 February 2023. *S. aureus* genomes from Malaysia were also included in the analysis. Other *S. aureus* genomes were chosen based on the availability of their complete genomes, but with more than 75,000 assembled genomes of *S. aureus* available in GenBank and more than 30,000 in PubMLST (as on 1 February 2023), these were picked more or less at random. The complete list of *S. aureus* strains used in phylogenetic analysis are provided in Supplementary Table S2. Pangenome analysis for *S. aureus* SauR3 and related *S. aureus* isolates listed in Table S2 was determined using ROARY, with the core genomes identified using the criteria of amino acid sequence identities of >95% [100] and presence in 99% of genomes. The derived core genome alignment was then used to infer a maximum-likelihood tree with 100 bootstraps under the generalized time reversible (GTR) model using FastTree [101,102]. The resulting phylogenetic tree was visualized using iTOL v6 (https://itol.embl.de/ accessed on 1 February 2023) [103].

## Data Availability

Sequences have been deposited in GenBank as follows: SauR3 chromosome (accession number CP098727), pSauR3-1 (accession number CP098728), pSauR3-2 (accession number CP098729), and pSauR3-3 (accession number CP098730).

## Supplementary Materials

The following supporting information can be downloaded at: https://www.mdpi.com/article/10.3390/pathogens12030502/s1, Table S1: List of genes encoding virulence factors detected in SauR3 genome; Table S2: List of *Staphylococcus aureus* strains used to construct the phylogenetic tree depicted in Figure 1.
