# Supplementary material for: Complete Genome Sequence and Analysis of a ST573 Multidrug-Resistant Methicillin-Resistant Staphylococcus aureus SauR3 Clinical Isolate from Terengganu, Malaysia
Source: Pathogens. 2023 Mar 22;12(3):502. doi: 10.3390/pathogens12030502 (PMC10053073; doi:10.3390/pathogens12030502)
Supplement: Supplementary file 1 [file pathogens-12-00502-s001.zip › Supplementary Table S1 & Table S2.pdf]

**Supplementary materials:**

# Complete Genome Sequence and Analysis of a ST573 Multidrug-Resistant Methicillin-Resistant *Staphylococcus aureus* SauR3 Clinical Isolate from Terengganu, Malaysia

Esra'a I. Al-Trad[1], Ainal Mardziah Che Hamzah[2], Suat Moi Puah[3], Kek Heng Chua[3], Muhamad Zarul Hanifah[4], Qasim Ayub[4], Prasit Palittapongarnpim[5], Stephen M. Kwong[6], Ching Hoong Chew[2*], and Chew Chieng Yeo[1*]

* Author for correspondence: chewch@unisza.edu.my (C.H.C.); chewchieng@gmail.com (C.C.Y.)

**Please refer Excel for supplementary tables,**
**Table S1:** List of genes encoding virulence factors detected in SauR3 genome.

**Table S2:** List of *Staphylococcus aureus* strains used to construct the phylogenetic tree depicted in Figure 1.
